# Total RNA and genomic DNA of *Lactobacillus gasseri* OLL2809 induce interleukin-12 production in the mouse macrophage cell line J774.1 via toll-like receptors 7 and 9

**DOI:** 10.1186/s12866-020-01900-w

**Published:** 2020-07-20

**Authors:** Kazumasa Onishi, Junko Mochizuki, Asako Sato, Ayako Goto, Toshihiro Sashihara

**Affiliations:** Food Microbiology Research Laboratories, R&D Division, Meiji Co., Ltd., 1-29-1 Nanakuni, Hachiouji, Tokyo, 192-0919 Japan

**Keywords:** *Lactobacillus gasseri* OLL2809, IL-12, Phagocytosis, Total RNA, Genomic DNA

## Abstract

**Background:**

*Lactobacillus gasseri* OLL2809 can highly induce interleukin (IL)-12 production in immune cells. Even though beneficial properties of this strain for both humans and animals have been reported, the mechanism by which the bacteria induces the production of IL-12 in immune cells remains elusive. In this study, we investigated the mechanism of induction of IL-12 using a mouse macrophage cell line J774.1.

**Results:**

Inhibition of phagocytosis of *L. gasseri* OLL2809, and myeloid differentiation factor 88 and Toll-like receptors (TLRs) 7 and 9 signalling attenuated IL-12 production in J774.1 cells. Total RNA and genomic DNA of *L. gasseri* OLL2809, when transferred to the J774.1 cells, also induced IL-12 production. The difference in the IL-12-inducing activity of *Lactobacilli* is attributed to the susceptibility to phagocytosis, but not to a difference in the total RNA and genomic DNA of each strain.

**Conclusion:**

We concluded that total RNA and genomic DNA of phagocytosed *L. gasseri* OLL2809 induce IL-12 production in J774.1 cell via TLRs 7 and 9, and the high IL-12-inducing activity of *L. gasseri* OLL2809 is due to its greater susceptibility to phagocytosis.

## Background

The health benefits of ingesting lactic acid bacteria in humans and animals are well known, and the protective effects of these bacteria in the intestinal environment [1] and in the context of allergy [2], hay fever [3], and obesity [4, 5] have also been reported. Some functional lactic acid bacteria are recognized as immunostimulants to the host immune cells, and confer the latter with high cytokine-inducing ability [6–8]. Further detailed studies have reported immune cells to recognize cell wall components [9] and nucleic acids [10–12] of some immunostimulant lactic acid bacteria via Toll-like receptors (TLRs), resulting in cytokine production. However, in many other functional lactic acid bacteria, the cytokine-inducing mechanism remains unclear.

*Lactobacillus gasseri* OLL2809 exhibits high interleukin (IL)-12-inducing ability [13]. IL-12 is a cytokine known to activate natural killer (NK) cells and induce the differentiation of naive T cells into T-helper 1 cells [14], thereby playing a vital role in the immune reaction. We have reported that *L. gasseri* OLL2809 exerts several immune-related effects on allergy [15, 16], endometriosis [17], NK activity after high-intensity exercise, and stress loading [18]. However, its IL-12-inducing mechanism in antigen-presenting cells such as macrophages has not yet been elucidated. In this study, we investigated the IL-12-inducing mechanism of *L. gasseri* OLL2809 in the murine macrophage J774.1 cell line addressing the question of the factors affecting the ability of lactic acid bacteria to induce cytokine production.

## Results

### Phagocytosis of *L. gasseri* OLL2809 by J774.1 cells

**Fig. 1:**
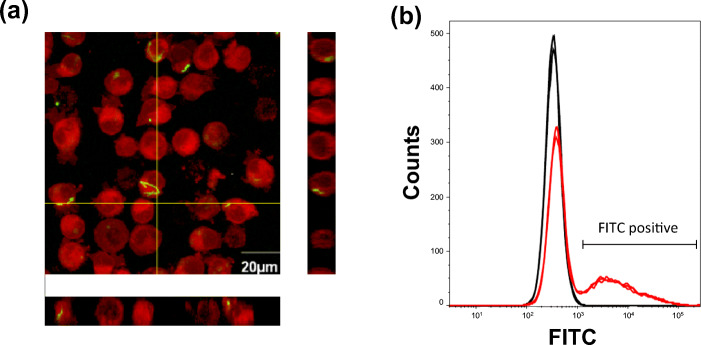
Confocal microscopic observation (**a**) and flow cytometric analysis (**b**) of J774.1 cells that phagocytosed *L. gasseri* OLL2809. After cultivation for 24 h in the presence of fluorescein-4-isothiocyanate (FITC)-labelled *L. gasseri* OLL2809 (green), J774.1 cells were labelled with Cell Mask™ Deep Red (red) and observed under a confocal microscope (**a**). The right and below panel showed x-z and y-z stacks at the yellow lines. After incubation for 48 h in the absence (black) or in the presence of fluorescein-4-isothiocyanate (FITC)-labelled *L. gasseri* OLL2809 (red), the J774.1 cells were labelled with allophycocyanin conjugated anti-F4/80 antibody and analysed by flow cytometry (**b**)

There have been several reports indicating that phagocytosis of lactic acid bacteria is required for initiation of IL-12 production from macrophages [9–11]. We first conducted a confocal microscopic analysis to clarify whether phagocytosis of *L. gasseri* OLL2809 by J774.1 cells is involved in an IL-12 production assay. It has been reported previously that stimulation of IL-12 production from J774.1 cells is more stable or even enhanced in heat-killed bacterial cells rather than live cells [13, 19]; therefore, heat-killed bacterial cells were used in the present study to eliminate the possible influence of probiotic properties such as viability or colonization of the live bacterial cells in the J774.1 culture. The results showed that the FITC-labelled *L. gasseri* OLL2809 were localized inside but not on the surface of several J774.1 cells (Fig. 1). A further flow cytometric analysis showed that about 30% of J774.1 cells were FITC positive. Considering the confocal microscopic observations and that flow cytometric analysis was performed after washing the J774.1 cells after labelling to remove excess dye and possible cell surface-attached, FITC-labeled *L. gasseri* OLL2809, these FITC-positive J774.1 cells are thought to be those that phagocytosed *L. gasseri* OLL2809.

### Phagocytosis of *L. gasseri* OLL2809 and IL-12 production by CyD-treated J774.1 cells

**Fig. 2:**
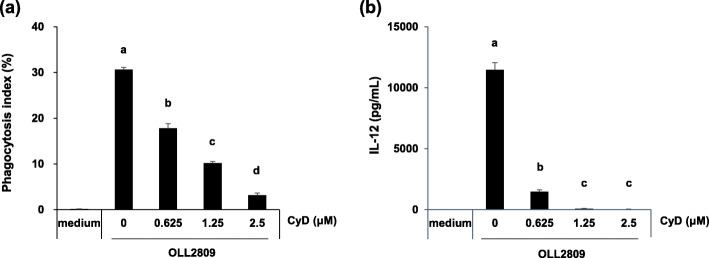
Effects of cytochalasin D treatment on phagocytosis and IL-12 production in J774.1 cells. J774.1 cells were pre-treated with cytochalasin D (CyD) for 1 h, followed by determination of phagocytosis index by flow cytometry (**a**) and IL-12 production assay (**b**). Data are expressed as mean with SD (*n* = 3). Difference between multiple groups was analysed by Tukey-Kramer multiple comparison test (**a**, **b**). a-d: different letters denote significant differences between the groups (*p* < 0.05)

Cell wall components and nucleic acids of lactic acid bacteria act as immunostimulants [9–12, 20], and the pattern-recognition receptors (PRRs) of immune cells that induce cytokines could be expressed both on the cell surface or inside the cells. Lipopolysaccharide (LPS), a ligand of gram-negative bacteria for cell-surface-expressed TLR4, stimulates IL-12 production without being phagocytosed [11]. On the other hand, several gram-positive bacterial cell wall components such as peptidoglycan, a ligand for cell-surface expressed TLR2, are also involved in the induction of IL-12 production from macrophages [21, 22]. To clarify whether stimulation of IL-12 production occurs without phagocytosis via cell-surface expressed PRRs or after phagocytosis followed by recognition of the immunostimulatory component of *L. gasseri* OLL2809 by intracellular PRRs, IL-12 production assay was performed after inhibition of phagocytosis using CyD. Stimulation of IL-12 production was inhibited by CyD in a concentration-dependent manner concomitant with a decrease in phagocytosis of the FITC-labelled *L. gasseri* OLL2809, as revealed by flow cytometry (Fig. 2a, b). Here, CyD treatment at 2.5 μM did not affect the cytotoxicity of the J774.1 cells as well as IL-12 production when stimulated with Pam3CSK4 [23], a synthetic bacterial lipopeptide and a known TLR2-TLR1 ligand (Figure A1). It is therefore suggested that receptors that recognize the components of *L. gasseri* OLL2809 and play an important role in IL-12 production are localized inside the cells rather than on the cell surface.

### TLRs 7 and 9 inhibition

**Fig. 3:**
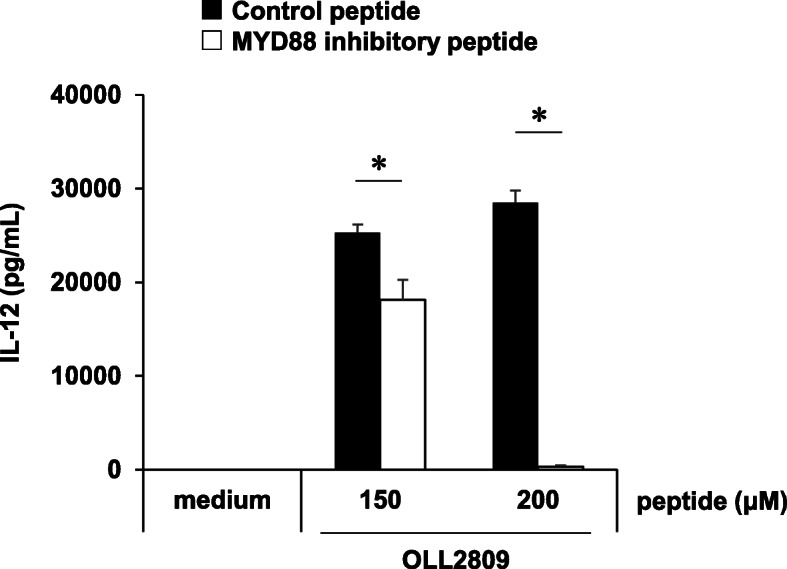
Effects of Myeloid differentiation factor 88 (MYD88) inhibitory peptide treatment on IL-12-inducing ability of *L. gasseri* OLL2809. J774.1 cells pre-treated with MYD88 inhibitory peptide followed by IL-12 production assay. (■; control peptide, □; MYD88 inhibitory peptide). Data are expressed as mean with SD (*n* = 3). Asterisk (*) represent a significant difference between groups (*p* < 0.05)

TLRs are well-known PRRs involved in cytokine production. It is known that TLRs 3, 7, and 9 are expressed in endosomes of immune cells, and that TLR3 signalling is MYD88-independent whereas TLR 7 and 9 signalling is MYD88-dependent [24]. To examine whether these TLRs of J774.1 cells are involved in IL-12 induction by *L. gasseri* OLL2809, MYD88 signalling pathways were inhibited in the IL-12 production assay. As shown in Fig. 3, the IL-12 concentration in the culture supernatant decreased depending on the concentration of the MYD88 inhibitory peptide, and no IL-12 production was observed when the cells were treated with 200 μM of the inhibitory peptide. Here, the viability of J774.1 cells was not affected by MYD88 inhibitory peptide (Figure A2).

**Fig. 4:**
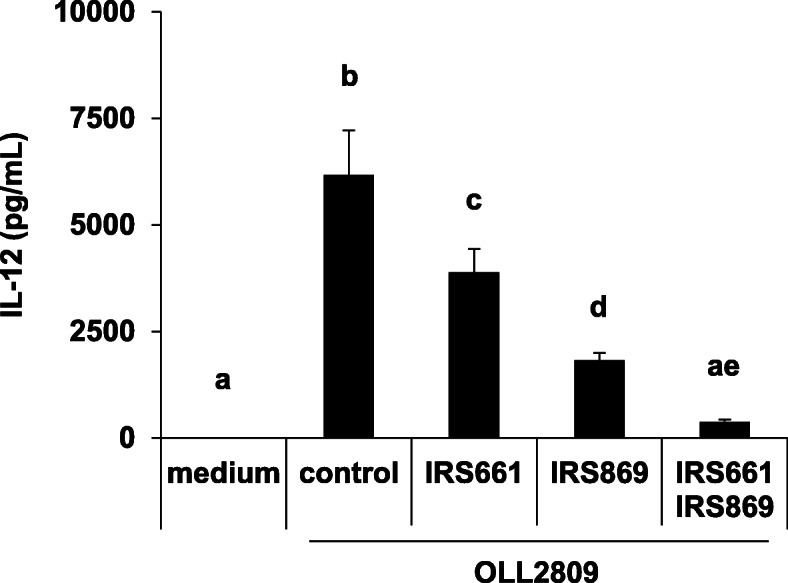
Effects of Toll-like receptors (TLRs) 7 and 9 antagonist pre-treatment on IL-12 production in J774.1 cells. J774.1 cells were pre-treated with control oligonucleotide (Control), IRS661, IRS869, or both the antagonists for 30 min, followed by IL-12 production assay. Data are expressed as mean with SD (*n* = 3). Difference between multiple groups was analysed by Tukey-Kramer multiple comparison test. a-e: different letters denote significant differences between the groups (*p* < 0.05)

When TLRs 7 or 9 were individually inhibited by IRS661 or IRS869, IL-12 production was significantly decreased as compared with control ODN. In addition, when both TLRs 7 and 9 were inhibited simultaneously, IL-12 production decreased to almost the same level as that in the group treated with medium (Fig. 4). Although the cell viability was significantly reduced by addition of 1 μg/mL of *L. gasseri* OLL2809, inhibition of TLRs 7 and 9 by these peptides was not found to be cytotoxic to J774.1 cells in this experimental condition (Figure A3). These results indicate that both TLRs 7 and 9 play an important role in IL-12 induction in response to *L. gasseri* OLL2809.

### IL-12 production by J774.1 cells treated with RNA and DNA of *L. gasseri* OLL2809

**Fig. 5:**
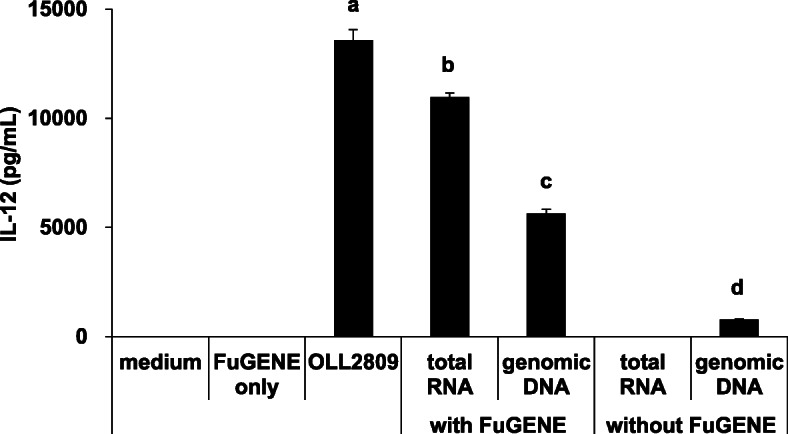
IL-12-inducing abilities of total RNA and genomic DNA derived from *L. gasseri* OLL2809. Total RNA and genomic DNA derived from *L. gasseri* OLL2809 were transferred to J774.1 cells, and then IL-12 production assay was performed. Data are expressed as mean with SD (*n* = 3). Difference between multiple groups was analysed by Tukey-Kramer multiple comparison test. a-d: different letters denote significant differences between the groups (*p* < 0.05)

It is known that the ligands of TLRs 7 and 9 are single-stranded RNA and unmethylated DNA, respectively [25, 26]. Therefore, IL-12-inducing abilities of total RNA and genomic DNA extracted from *L. gasseri* OLL2809 were investigated. Both total RNA and genomic DNA of *L. gasseri* OLL2809 transferred to J774.1 cells with FuGENE induced IL-12 production, although there were significant differences in the intensities among the stimulants (Fig. 5). These effects were not observed without the transfection reagent. These findings suggest that the total RNA and genomic DNA of *L. gasseri* OLL2809 were recognized by receptors localized inside cells.

### Relationship among the phagocytosis index and IL-12 induction ability of *Lactobacillus* strains, and IL-12-inducing abilities of their total RNA and genomic DNA

**Fig. 6:**
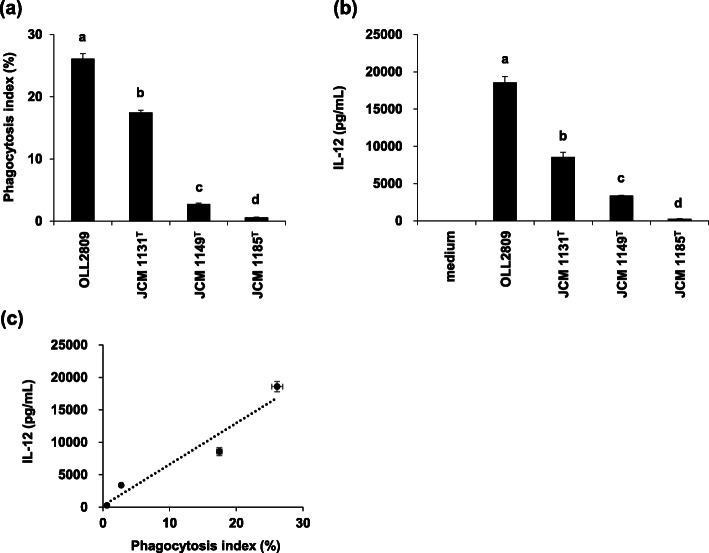
Relationship between phagocytosis index and IL-12-inducing abilities of various *Lactobacilli* strains. J774.1 cells were cultivated in the presence of FITC-labelled *L. gasseri* OLL2809, *L. gasseri* JCM 1131^T^, *L. plantarum* JCM 1149^T^, and *L. crispatus* JCM 1185^T^, respectively. After incubation for 48 h, J774.1 cells were labelled with APC conjugated anti-F4/80 antibody and phagocytosis index was analysed by flow cytometry (**a**). The concentration of IL-12 in the culture supernatant was quantified by ELISA (**b**). The IL-12 levels and phagocytosis index of each strain are plotted. Pearson correlation coefficient; *r* = 0.0666, *n* = 4, *p* < 0.05 (**c**). Data are expressed as mean with SD (*n* = 3). Difference between multiple groups was analysed by Tukey-Kramer multiple comparison test (**a**,**b**). **a**-**d**: different letters denote significant differences between the groups (*p* < 0.05)

**Fig. 7:**
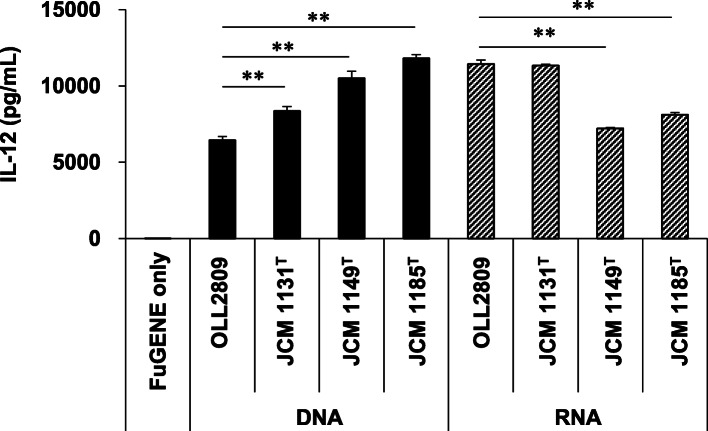
IL-12-inducing abilities of total RNA and genomic DNA derived from *Lactobacilli* strains. Total RNA and genomic DNA derived from *L. gasseri* OLL2809, *L. gasseri* JCM 1131^T^, *L. plantarum* JCM 1149^T^, and *L. crispatus* JCM 1185^T^ were transfected to J774.1 cells using FuGENE, and IL-12 production assay was performed. Data are expressed as mean with SD (*n* = 3). Difference between multiple groups was analysed by Dunnett’s multiple comparison test, in which genomic DNA and total RNA were analysed independently. **: *p* < 0.01 vs either of the genomic DNA or total RNA isolated from *L. gasseri* OLL2809

We have reported that immunostimulatory activities are species- and strain-dependent [13]. To examine whether the difference is due to phagocytosis or bacterial cell component for TLR signalling, phagocytosis index, and IL-12-inducing ability of *L. gasseri* OLL2809, *L. gasseri* JCM 1131^T^, *L. plantarum* JCM 1149^T^ and *L. crispatus* JCM 1185^T^ were analysed. As shown in Fig. 6, a significant positive correlation was found between phagocytosis index and IL-12-inducing ability (Pearson correlation coefficient; *r* = 0.0666, *n* = 4, *p* < 0.05). Furthermore, in order to evaluate the influence of differences in nucleotide sequences on the IL-12-inducing ability, total RNA and genomic DNA extracted from these lactic acid bacterial cells were transferred to J774.1 cells. The results showed that IL-12 production was substantially induced in all groups including those derived from strains that exhibited less IL-12-inducing activity such as *L. plantarum* JCM 1149^T^ and *L. crispatus* JCM 1185^T^, although the activity of total RNA extracted from these strains was slightly low but with statistical significance when compared with that of *L. gasseri* OLL2809 (*p* < 0.001, Fig. 7). These data indicate that susceptibility to phagocytosis by J774.1 cells is the most important factor for the IL-12-inducing ability of lactic acid bacteria.

## Discussion

There are roughly two routes by which immune cells recognize microorganisms and induce inflammatory cytokines for self-defence against their infection: recognition by PRRs expressed on the cell surface or by intracellular organs. These receptors, expressed on both the cell surface and inside the cells, play an important role in the recognition of lactic acid bacteria, and the cytokine induction in the immune cells [11]. CyD, a phagocytosis inhibitor, was used to verify whether *L. gasseri* OLL2809, which was confirmed to be phagocytized (Fig. 1), is recognized by either of the extracellular and intracellular receptors of J774.1 cells. Phagocytosis by J774.1 cells was inhibited by CyD, and IL-12 production was markedly suppressed concomitant with a decrease in phagocytosis of *L. gasseri* OLL2809 (Fig. 2). This suggested that IL-12 production by J774.1 cells was induced via the receptor expressed on the intracellular organ after phagocytosing *L. gasseri* OLL2809. To investigate whether TLRs, one of the most studied PRRs in immune cells, are involved in the IL-12 induction by *L. gasseri* OLL2809, we focused on MYD88, an adapter molecule involved in signal transduction by various TLRs. It is known that TLRs 3, 7, and 9 are expressed in endosomes of immune cells, and that TLR3 signalling is MYD88-independent whereas TLRs 7 and 9 signalling are MYD88-dependent [24]. In our study, inhibition of MYD88 resulted in less production of IL-12 against *L. gasseri* OLL2809 stimulation (Fig. 3), suggesting that J774.1 cells recognized components of *L. gasseri* OLL2809 via TLRs 7 and/or 9 to induce production of IL-12. To clarify whether TLRs involved in IL-12 production were either or both of TLRs 7 and 9, antagonists of TLR7 and 9, IRS661 and IRS869, respectively, were used. *L. gasseri* OLL2809 was added to J 774.1 cells treated with either or both of IRS661 and IRS869. IL-12 production decreased when either TLR 7 or 9 was inhibited, and IL-12 production reduced to almost the same level as that in the control group when both TLRs 7 and 9 were simultaneously inhibited (Fig. 4). These results indicated that both TLRs 7 and 9 play an important role in IL-12 induction in response to *L. gasseri* OLL2809. It is known that the ligands of TLRs 7 and 9 are single-stranded RNA and unmethylated DNA, respectively [25, 26]. To further verify whether RNA and DNA of *L. gasseri* OLL2809 induce IL-12 production in J774.1, the IL-12-inducing ability of total RNA and genomic DNA of *L. gasseri* OLL2809 was examined. IL-12-inducing abilities were observed for both total RNA and genomic DNA of *L. gasseri* OLL2809 when transferred using a FuGENE system (Fig. 5).

In addition, we clarified a positive correlation between the phagocytosis index and IL-12 inducing-ability using different *Lactobacillus* strains, i.e.*,* the IL-12-inducing ability was higher in strains more susceptible to phagocytosis (Fig. 6). It was also found that IL-12 production was uniformly induced in J774.1 cells when total RNA or genomic DNA derived from *Lactobacillus* strains that exhibit less IL-12-inducing activity was transferred (Fig. 7).

In this study, we demonstrated that phagocytosis and the TLR 7 and 9 signalling pathway are involved in the IL-12-inducing mechanism of *L. gasseri* OLL2809 in J774.1 cells. We further showed that the susceptibility to phagocytosis is highly correlated with the IL-12-inducing ability of different lactic acid bacterial strains. Our findings that both genomic DNA and total RNA of various strains exhibited substantial IL-12 inducing ability, although the activities are slightly different in various strains, suggest that phagocytosis is a more crucial factor determining the IL-12 inducing activity of different lactic acid bacterial strains than the actual immune stimulants.

## Conclusion

We concluded that total RNA and genomic DNA of phagocytosed *L. gasseri* OLL2809 induce IL-12 production in J774.1 cell via TLRs 7 and 9, and the high IL-12-inducing activity of *L. gasseri* OLL2809 is due to its greater susceptibility to phagocytosis. Further studies are required to identify the components of lactic acid bacteria that mediate their phagocytosis by immune cells, and the receptors of the macrophage cells that play an important role in phagocytosis to clarify the mechanism by which different lactic acid bacteria exert different immune-modulatory effects.

## Methods

### Bacterial strains and growth conditions

*L. gasseri* OLL2809 isolated in our laboratory as described previously [13] was cultured in BD Difco™ Lactobacilli MRS Broth (Thermo Fisher Scientific, Waltham, MA, USA) at 37 °C for 18 h. After fermentation, the bacterial cells were harvested in a refrigerated centrifuge at 10,000×*g* for 15 min and washed twice with saline solution followed by one wash with distilled water. The cells were then resuspended in distilled water, heated at 75 °C for 1 h, and lyophilized. The lyophilized cells were resuspended in phosphate-buffered saline (PBS; pH 7.4) at a concentration of 200 μg/ml and used for IL-12 production assays.

*L. gasseri* JCM 1131^T^, *L. plantarum* JCM 1149^T^ and *L. crispatus* JCM 1185^T^ were purchased from the Riken BRC (Wako, Japan). They were cultured, heat treated and lyophilized as described above.

### J774.1 cell culture

The murine macrophage cell line J774.1 was purchased from the Riken BRC (Wako, Japan). J774.1 cells were cultured in Roswell Park Memorial Institute medium 1640 (RPMI 1640; Thermo Fisher Scientific) supplemented with 10% fetal bovine serum (FBS; Biosera, Nuaille, France), 100 U/mL penicillin (Thermo Fisher Scientific), 100 μg/mL streptomycin (Thermo Fisher Scientific) and 55 μM 2-mercaptoethanol (Thermo Fisher Scientific) in a humidified 5% CO_2_ incubator at 37 °C.

### IL-12 production assay

J774.1 cells were seeded at 5 × 10^4^ cells per 100 μL per well in a 96-well plate and cultivated for 48 h in the absence or presence of 1 μg/ml of the *L. gasseri* OLL2809. The IL-12 (p40) levels in the culture supernatant were quantified using the mouse IL-12(p40) ELISA set (BD Biosciences, Franklin Lakes, NJ, USA). The same cultivation conditions were used for all experiments, if not otherwise specified.

### Fluorescein-4-isothiocyanate (FITC) labelling and confocal microscopy

Lyophilized *L. gasseri* OLL2809 (10 mg) were resuspended in 1 mL of 50 mM carbonate buffer (pH 9.6) containing 5 μg FITC (Dojindo, Kumamoto, Japan), and incubated at 37 °C for 1 h. After washing twice with PBS (pH 7.4), the cells were used as FITC-labelled *L. gasseri* OLL2809. J774.1 cells were cultivated with 2 μg/mL FITC-labelled *L. gasseri* OLL2809 for 24 h. They were then stained with Cell Mask™ Deep Red (Thermo Fisher Scientific) and observed with confocal microscopy (Olympus, Tokyo, Japan).

### Flow cytometric analysis

J774.1 cells were cultivated in the presence of 2 μg/mL FITC-labelled *L. gasseri* OLL2809 for 48 h. They were then harvested and labelled with allophycocyanin (APC) conjugated anti-F4/80 antibody (Bio-Rad, Hercules, CA, USA) and 7-amino-actinomycin D (7-AAD; BD Biosciences). The J774.1 cells were washed 2 twice with PBS (pH 7.4) and 7-AAD-negative, FITC-positive and F4/80-positive cells were counted on the flow cytometer FACSVerse™ (BD Biosciences). The proportion of FITC-positive cells in F4/80-positive cells was defined as phagocytosis index, and that of 7-AAD- positive cells was defined as the dead cell ratio.

### Inhibition of phagocytosis, and MYD88 and TLRs 7 and 9 signalling

For inhibition of phagocytosis and Myeloid differentiation factor 88 (MYD88) and TLR 7 and 9 signalling, J774.1 cells were treated with various reagents followed by IL-12 production assays.

For phagocytosis, J774.1 cells were pre-treated with 0, 0.625, 1.25, 2.5 μM cytochalasin D (CyD; Fujifilm Wako Pure Chemical, Osaka, Japan) for 1 h. For MYD88 signalling, J774.1 cells were pre-treated with 150, 200 μM of MYD88 homodimerization inhibitory peptide or the control peptide (Novus Biologicals, Centennial, CO, USA) for 24 h. The culture medium was replenished with fresh media, and then IL-12 production assay was performed. The amino acid sequences of the peptides were as follows;

Control peptide: DRQIKIWFQNRRMKWKK

Inhibitory peptide: DRQIKIWFQNRRMKWKKRDVLPGT

For TLRs 7 and 9, TLR7 antagonist immunoregulatory sequence (IRS)661 [27], and TLR9 antagonist IRS869 [10] as well as the control oligodeoxynucleotide (ODN) [28], were synthesized by Eurofins Genomics (Tokyo, Japan). J774.1 cells were pre-treated with 1 μM of IRS661, or IRS869, or both for 30 min. Nucleotide sequences of ODNs were as follows;

Control: TCCTGCAGGTTAAGT

IRS661 (Antagonist of TLR7): TGCTTGCAAGCTTGCAAGCA

IRS869 (Antagonist of TLR9): TCCTGGAGGGGTTGT

### Extraction of RNA and DNA from bacterial strains and transfer to J774.1 cells

Total RNA and genomic DNA were extracted from bacterial strains by using Nucleo Spin® RNA (Takara Bio, Shiga, Japan) or Nucleo Spin® Microbial DNA (Takara Bio). The concentration of the extracted nucleotides was measured by Nano Drop 1000 (Thermo Fisher Scientific). Further, 5 μL of FuGENE® HD (Promega, Fitchburg, WI, USA) was added to 100 μL of Opti-MEM (Thermo Fisher Scientific) containing 2 μg of the extracted nucleotide, and the mixtures were incubated for 10 min at room temperature to form total RNA/FuGENE or genomic DNA/FuGENE complexes. The total RNA/FuGENE or genomic DNA/FuGENE were diluted 20-fold and used to stimulate J774.1 cells for IL-12 production assays.

### Statistical analyses

Data are expressed as mean values with standard deviations (SD). Difference between multiple groups was analysed by Tukey-Kramer or Dunnett’s multiple comparison test. For analysis of the two groups in the MYD88 signal inhibition experiment, the Student’s *t*-test was used. Differences were considered significant when the *p*-values for the effect were less than 0.05.

## Supplementary information

**Additional file 1: Figure A1.** Effects of cytochalasin D treatment on viability of J774.1 cells (a) and IL-12 production stimulated with Pam3CSK4. **Figure A2.** Viability of J774.1 cells during MYD88 inhibition assay. **Figure A3.** Viability of J774.1 cells during TLRs 7 and 9 antagonist assay. (XLS 294 kb)

## Data Availability

The data set supporting our results are included within the article.

## Abbreviations

IL: Interleukin
TLR: Toll-like receptor
NK: Natural killer
RPMI: Roswell Park Memorial Institute
FBS: Foetal bovine serum
FITC: Fluorescein-4-isothyocyanate
PBS: Phosphate-buffered saline
APC: Allophycocyanin
7-AAD: 7-amino-actinomycin D
MYD88: Myeloid differentiation factor 88
CyD: Cytochalasin D
IRS: Immunoregulatory sequence
ODN: Oligodeoxynucleotide
SD: Standard deviation
PRR: Pattern-recognition receptor
LPS: Lipopolysaccharide
